# Impact of mutations in starch synthesis genes on morphological, compositional, molecular structure, and functional properties of potato starch

**DOI:** 10.1371/journal.pone.0310990

**Published:** 2024-09-26

**Authors:** Shishanthi Jayarathna, Zsuzsanna Péter-Szabó, Gustav Nestor, Mariette Andersson, Francisco Vilaplana, Roger Andersson

**Affiliations:** 1 Department of Molecular Sciences, BioCenter, Swedish University of Agricultural Sciences, Uppsala, Sweden; 2 Division of Glycoscience, Department of Chemistry, KTH-Royal Institute of Technology, AlbaNova University Centre, Stockholm, Sweden; 3 Department of Plant Breeding, Swedish University of Agricultural Sciences, Lomma, Sweden; University of Delhi, INDIA

## Abstract

Morphology, composition and molecular structure of starch directly affect the functional properties. This study investigated the morphological, compositional, and molecular structure properties of starch from starch branching enzyme gene (*SBE*) and granule-bound starch synthase gene (*GBSS*) mutated potato, and their associations with thermal, pasting, and film-making properties. *SBE* mutations were induced in native variety Desiree while *GBSS* mutations were herestacked to a selected *SBE* mutated parental line. Mutations in *SBE* resulted in smaller starch granules and higher amylose content, while *GBSS* mutations in the *SBE* background reduced amylose content. Mutations in *SBE*, particularly with *GBSS* mutations, significantly increased total phosphorus content. ^31^P NMR spectroscopy revealed higher proportions of C6-bound phosphate than of C3-bound phosphate in all studied lines. Amylopectin unit chain and internal chain distributions showed higher proportions of long chains in mutated lines compared with Desiree. These amylopectin long-chains were positively correlated with gelatinizationand, pasting temperatures, and temperature at peak viscosity. Short amylopectin chains showed positive correlations with breakdown viscosity, but negative correlations with the crystal melting temperature of retrograded starch. Total phosphorus content was positively correlated with the crystal melting temperature of retrograded starch. Starch from different lines was used to produce a series of potato starch films that differed in morphology and functional properties. A negative correlation was observed between Young’s modulus of films and the long amylopectin-chain fraction. Thermal gravimetric analysis revealed highest thermal stability of Desiree starch films, followed by films from *SBE*-mutated high-amylose lines. Oxygen transmission rate and oxygen permeability analyses showed that films made with starch from selected *GBSS* and *SBE*s mutated line maintained comparable oxygen barrier properties to Desiree film. These insights on the impact of genetic mutations on starch properties indicate potential applications of *in-planta* starch modification for specific end-uses including packaging.

## 1. Introduction

Environmental pollution arising from petroleum-based plastics is a significant global problem. Transitioning towards bioplastics, especially for packaging applications, is a promising strategy for addressing this issue. Among the polymers utilized in bioplastic manufacturing, starch has the advantages of being renewable, biodegradable, non-toxic, abundant, and cost-effective. However, inherent drawbacks of starch-based bioplastic materials, such as inferior mechanical properties and susceptibility to water, prevent widespread utilization of starch in its native state. Several strategies can be employed to overcome issues associated with use of starch in bioplastic applications. These include post-harvest starch modification through physical, chemical, enzymatic, or combined methods, blending with alternative polymers, incorporating compatibilizers and/or reinforcing agents, and *in-planta* starch modification. Of these, *in-planta* starch modification is the most sustainable method due to its avoidance of chemicals or energy use [1].

The functional properties of starch are governed by the composition and molecular structural characteristics of its main macromolecular components, amylose and amylopectin. Amylose is distinguished by its predominantly linear (1→4)-linked α-glucan chains, which can achieve degree of polymerisation (DP) as high as 600. Amylopectin comprises (1→4)-linked α-glucan chains with α-(1→6) branch points and is the predominant fraction in native starch granule [2]. Some progress has been made in *in-planta* modification of the composition and/or molecular structure, granule size, and phosphorylation of starch to suit specific end-uses. For instance, in the 1950s amylose was identified as a molecule with good film-forming ability, and potential of amylose films in industrial applications was suggested [3]. Since then, various attempts have been made to produce amylose-rich or pure amylose starch *in-planta*, and to assess amylose for its film-forming behaviour [4–8]. Starch granule size is a crucial factor for industrial applications, prompting efforts to tailor granule size to match specific end-use bioplastic applications. For example, Ji et al. [9] used genetic engineering approaches to produce smaller potato starch granules with particular potential in starch films. Another significant aspect of starch governing its functionality and with potential for *in-planta* modification is phosphorylation. Potato starch exhibits a higher degree of phosphorylation than cereal starches, contributing significantly to its functional properties. Utilizing transgenic approaches to achieve a specific level of phosphorylation in potato starch for film formulations has demonstrated promise in producing starch films with desired properties [10].

The physicochemical properties of starch cannot always be accurately anticipated by analysing individual molecular parameters in isolation [11]. The properties of starch, in combination, determine its functional characteristics. Therefore, a holistic approach is required to identify granular, molecular, and compositional features, and interactions between these, determining the functional properties of starches. In this study, we focused on starch from potato lines with the same genetic background, but with induced mutations in genes encoding key enzymes of the starch synthesis pathway, with the aim of uncovering how specific mutations affect molecular, granular, and compositional traits, and starch thermal, pasting, and film-making properties. Our starting hypothesis was that various precise mutations in starch synthesis genes can induce alterations in the molecular, granular, and compositional features of the starch, thereby affecting film formation and functional properties. To the best of our knowledge, this study is the first to explore the potential for utilizing CRISPR/Cas9 technology to develop different starch qualities for film applications. The unique findings obtained can be important in guiding the design of tailor-made starches for specific end-use applications, by establishing the links between genetic background, granular and molecular features of starch, and different functional properties.

## 2. Materials and methods

### 2.1. Potato starch samples

All eight potato starch types studied (see Table 1) were provided by the Department of Plant Breeding, Swedish University of Agricultural Sciences, Alnarp, Sweden. The four potato lines starting with 1040 were developed by CRISPR/CAS9 mutagenesis of *SBEI* and *II* [12]. The three potato lines starting with 150 were developed by stacking mutations in the *GBSS* gene to an *SBE*s mutated background [13]. All potato lines were cultivated in the greenhouse between 19 August and 14 December 2022, under controlled conditions as described elsewhere [12]. Starch was isolated according to Larsson et al. [14]. The specific mutations of the different potato lines are indicated in Table 1.

**Table 1:** Size of induced mutations in potato genes *SBEI*, *SBEII*, *and GBSS*.

Breeder’s ID code	Genetic background	*SBEI*	*SBEII*	*GBSS*
		(size of indels)	(size of indels)	(size of indels)
104006	Desiree	-92;-5;-1	-11;-1;0	NA
104016	Desiree	-93;-4	-2;0;+6	NA
104018	Desiree	-93;-23;-17;+153	-1;0;+104	NA
104034	Desiree	-94;-93;-5;+60	-8;-6;0	NA
150172	104018	-93;-23;-17;+153	-1;0;+104	-3;-2;-2;+1
150183	104018	-93;-23;-17;+153	-1;0;+104	-37;-7;-5;-3
150068	104018	-93;-23;-17;+153	-1;0;+104	-5;-5;-1;-1
Desiree	Native variety	NA	NA	NA

“-” represents deletion, “+” represents insert, and lines with a “0” have at least one wild-type allele. Where only three different sizes of indels are shown, at least two alleles have indels of the same size. NA: no mutations in the particular gene.

### 2.2 Characterisation of isolated potato starches

#### 2.2.1 Analysis of starch particle size

The particle size distribution of the starches was studied using a SALD-2300 laser diffraction particle size analyser with SALD-BC23 batch cell system configuration (Shimadzu, Japan). Starch was dispersed in ethanol and homogenized before the measurements. The operating environment comprised temperature maintained between 10 and 30 °C at 20–80% humidity. Mean particle size of isolated starch granules was determined using the Wing SALD II software. The analysis was performed in triplicates and results are presented as mean of triplicates.

#### 2.2.2 Analysis of amylose content

Amylose content in the starches was measured using the assay protocol outlined in the amylose/amylopectin kit from Megazyme, Wicklow, Ireland. The amylopectin fraction was first precipitated using Concanavalin A, and then the supernatant with amylose was enzymatically hydrolyzed to D-glucose and analysed using glucose oxidase/peroxidase reagent. The analysis was conducted in duplicate, and values reported are mean of these duplicates.

#### 2.2.3 Analysis of total phosphorus content and different forms of phosphorus

The phosphorus content in starch was determined using a Spectro Blue inductively coupled plasma instrument (Spectro Analytical Instruments, Germany) with a modified SS 028311 method. Samples were weighed to precision of 2–4 decimal places using a glass vial. Each glass vial was then filled with 20 mL of a mixture comprising 50% concentrated nitric acid and 50% deionized distilled water, and the vials were heated on a heat block for slightly over one hour until simmering. Following cooling, the vials were topped up with deionized distilled water to a final volume of 100 mL and thoroughly mixed, filtered, and centrifuged. The particle-free liquid was then analysed for phoshorus.

Nuclear magnetic resonance (NMR) spectroscopy was used to determine the absolute amounts of different forms of phosphorus in the starch samples. Starch suspensions in water (0.1g/10 mL) were degraded with thermostable α-amylase from *Bacillus* sp. (EC 3.2.1.1, ∼170 U/mg; Megazyme, Wicklow, Ireland) for full dissolution. The samples were then subjected to dual freeze-drying and re-dissolved in D_2_O before final dissolution in 600 μL D_2_O. Next, EDTA (0.5 M in D_2_O, pH 8.0) was added to obtain an EDTA sample concentration of 25 mM, for enhancement of spectral quality and resolution, and sample pH was adjusted to 8.0 (equivalent to pD 8.5) with sodium hydroxide (0.1 M in D_2_O). ^31^P NMR spectra were recorded at 25°C on a Bruker Avance III 600 MHz spectrometer with a 5 mm broadband observe detection SmartProbe. Spectra were acquired with inverse gated ^1^H decoupling and 256 scans, using a spectral width of 8 ppm, an acquisition time of 2.1 s, and a relaxation delay of 5 s. To confirm spectral assignments, ^31^P spectra without ^1^H decoupling were recorded, as well as ^1^H,^31^P-HMBC and ^31^P-DOSY spectra. The ^31^P spectra were processed with TopSpin 4.1.4 to calculate relative amounts of various phosphorus forms by integration of the corresponding NMR signals. Absolute amounts were then calculated from the total phosphorus content analysed by the inductively coupled plasma technique. ^31^P chemical shifts were indirectly referenced to external phosphoric acid (85%).

#### 2.2.4 Analysis of molecular structure

The chain length distribution pattern of de-branched whole starch and the internal chain distribution were investigated in duplicates. For analysis of de-branched chain length distribution, whole starch samples were de-branched using isoamylase from *Pseudomonas* sp. (EC 3.2.1.68, 500 U/mL; Megazyme, Wicklow, Ireland) and pullulanase M1 from *Klebsiella planticola* (EC 3.2.1.41, 700 U/mL; Megazyme, Wicklow, Ireland), after solubilizing in UDMSO (0.6 M urea in 90% DMSO), following the exact protocol outlined in Jayarathna et al. [13]. The resulting de-branched starch samples were analysed by high-performance size exclusion chromatography (HPSEC), following a methodology described before [13]. A five-fold diluted sample preparation from HPSEC was then used in high-performance anion-exchange chromatography (HPAEC) analysis. The HPAEC instrumentation comprised an ICS-6000 series device from Dionex Corp. (Sunnyvale, CA, USA), coupled with a pulsed amperometric detector (PAD). Instrumental settings and programs were as described elsewhere [13].

Internal chain distribution was investigated using HPSEC on beta-limit dextrins (β-LD) obtained by treating whole starch with barley β-amylase and then de-branching, as described before [15]. The HPSEC setting was similar to that used in de-branched whole starch chain distribution analysis.

#### 2.2.5 Analysis of pasting properties

Starch pasting properties were examined using a Discovery HR-3 hybrid rheometer (TA Instruments, New Castle, DE, USA), following the protocol outlined by [16] with some modifications. In brief, starch dispersions containing 2.08 g starch in 26 mL distilled water were subjected to a heating-cooling cycle (30 to 130 to 38 °C) inside a Peltier pressure cell equipped with a steel paddle. The heating rate was set at 5°C per minute, while the cooling rate was 4.2°C per minute. Prior to the cooling cycle, the samples were held at 130°C for 5 minutes. The rotational speed initially reached the device maximum (50 rad/s) for the initial 20 s, and was then maintained at 16.75 rad/s throughout the experiment. All analyses were performed in duplicate and results are presented as mean of duplicates.

#### 2.2.6 Analysis of thermal properties

Starch gelatinization properties and retrogradation properties were analysed by differential scanning calorimetry (DSC) with a DSC250 device (TA Instruments, New Castle, DE, USA) calibrated with indium. Gelatinization onset, peak, and end temperatures, and the associated enthalpy change, were investigated in duplicates following the methodology described by Zhao, Hofvander, Andersson, & Andersson (2023) [17]. For retrogradation studies, the same DSC pans used for the gelatinization experiment were directly stored in a refrigerator at 5°C for 3 days. The samples then underwent a heating cycle from -5 to 120 °C, with a heating rate of 10°C/min and an initial temperature of 5°C. The peak temperature of melting of retrograded starch and the associated enthalpy change were then determined.

### 2.3 Starch film formulation and characterisation

Starch films were prepared following the method described by Menzel et al. [4] with slight modifications. In summary, starch dispersions of 180 mg dry weight in 6 mL of water were heated at 128°C in a sealed tube for 45 minutes with continuous stirring using a Pierce Reacti-Therm heating/stirring module. The solutions were cooled to approximately 95°C, mixed with 40 μL glycerol for 5 minutes, and cast in 8.5 cm diameter Petri dishes (volume 8.4 mL). The solvent was allowed to evaporate overnight at 23°C, followed by a drying period of 48 hours at the same temperature. The films were characterized in terms of their optical, mechanical, and thermal properties, as outlined below.

#### 2.3.1. Morphological characterisation of starch film surface

Surface morphology of the starch films was characterized by acquisition of digital images and scanning electron microscopy (SEM) imaging conducted using an environmental electron microscope (Flex SEM 1000 II, Hitachi, Japan). Film samples measuring approximately 5 mm x 5 mm were affixed to an observation holder using double-sided tape, without application of a gold coating. Micrographs were captured for each starch film sample at magnification 100×.

#### 2.3.2 Evaluation of film mechanical properties

Tensile properties of different films were evaluated using a modified testing protocol based on ASTM D882-12 and ISO 527–1:2019 standards. The cast and dried films were conditioned in Petri dishes for 7 days before testing in a climate-controlled room at 23 ± 2 °C and 50 ± 10% relative humidity (RH). The samples were cut into specimens 10 mm wide and 80 mm long. The thickness of each sample was measured with a caliper at five points along its length, to ensure uniform cross-section values. Tensile testing was performed on five specimens from each of the two different preparations (batches) of films. The initial gauge length for the measurements was 25 mm and the tests were conducted with a 0.1 min^-1^ strain rate.

#### 2.3.3 Thermal property characterisation of films

Thermal degradation of the starch films was investigated by thermogravimetric analysis (TGA), using a Mettler-Toledo TGA/DSC 1 instrument. Approximately 6 mg of sample were heated at a rate of 10 °C/min in a range from 30 to 600 °C, under nitrogen flow of 50 mL/min. TGA was performed on three specimens from each of the two different preparations (batches) of films. The weight loss corresponding to different degradation steps was determined from the thermographs. The first derivative TGA curves (DTG) were used to identify the inflection points where weight loss occurred with the highest velocity.

#### 2.3.4 Evaluation of gas barrier properties

Gas barrier properties of the film from potato line 150068 starch compared with film from the native variety (Desiree) starch were evaluated using an Ox-Tran 2/21 SH instrument (AMETEK-MOCON, Minneapolis, MN, USA) following ASTM-F1927 standard procedure. Oxygen permeability and oxygen transmission rate were measured in a 5 cm^2^ exposure area of duplicate films after pre-conditioning at 23°C, 50% RH for 10 hours.

### 2.4. Statistical analysis

Differences in measured parameters were studied by one-way analysis of variance (ANOVA). Tukey pairwise comparisons and Pearson correlation coefficient analysis were performed at confidence level 95% (p < 0.05) using Minitab 21 software (State College, PA, USA).

## 3. Results and discussion

### 3.1. Properties of granular starch

#### 3.1.1 Starch granule size

The granule size of starch from mutated lines was measured and compared with that of the native variety (Desiree) (Table 2). The granule size of the native variety was notably smaller (33 μm) than that reported for other native potato starch types (50–62 μm) [18]. This difference may arise from various environmental and biological factors influencing starch granule size. Environmental aspects such as temperature, drought, and fertilizer regime [19] and growth conditions such as pot size [20] contribute to variations in starch granule sizes even in the same variety grown on different occasions.

**Table 2:** Granule size, amylose content, total phosphorus (P) content, and absolute amounts of different forms of phosphorus in various potato starch samples.

Potato line	Granule size D[4,3] (μm±2.5)	Amylose content (%) (±0.6)	Total P content (g/kg)	C3^1^	C6^2^	C6^3^	C3/C6	Proportion of total starch-attached phosphates	Free Phosphates (g/ kg)	Phospholipids (g/ kg)
				(g/ kg)	(g/ kg)	(g/ kg)	(g/ kg)			
104006	24.3^g^±0.06	34^b^±0.2	2.2	0.33	0.73	0.69	0.23	0.79	0.34	0.12
104016	27.5^f^±0.04	29 ^c^±0.7	1.6	0.24	0.54	0.47	0.24	0.78	0.24	0.11
104018	22.0^h^±0.02	45^a^0.6	2	0.25	0.80	0.40	0.21	0.73	0.41	0.14
104034	29.2^d^±0.14	27^c^±0.9	1.7	0.24	0.72	0.40	0.21	0.79	0.24	0.11
150172	28.4^e^±0.2	10^e^±0.1	3	0.46	1.04	0.92	0.23	0.80	0.48	0.10
150183	33.4^a^±0.09	7^f^±0.7	2.8	0.49	0.87	0.90	0.28	0.81	0.42	0.12
150068	30.2^c^±0.2	23^d^±0.2	1.1	0.15	0.35	0.32	0.22	0.75	0.18	0.09
Desiree	32.5^b^±0.2	27^c^±0.8	0.6	0.10	0.17	0.14	0.33	0.69	0.08	0.10

Values within columns with different superscript letters differ significantly (ANOVA, α = 0.05). Values following the mean represent the standard deviation (SD)

^1^C3 substituted phosphate monoester.

^2^C6 substituted phosphate monoester type 1.

^3^C6 substituted phosphate monoester type 2.

Granule size was mainly similar to that of Desiree starch in all starch types from the mutated lines except 104006 and 104018, where granule size was smaller. These two potato lines possess similar mutation types, with at least two out of-frame mutations in *SBE II*, whereas in lines 104016 and 104034 at least one of the mutated alleles has an in-frame deletion. Notably, lines 104016 and 104034 did not demonstrate a pattern of decreasing starch granule size with *SBE* mutations. The reduction in starch granule size in the *SBE*-mutated high-amylose lines 104006 and 104018 aligns with findings from previous studies on starch from SBE-inhibited or mutated potato lines [18, 21]. It indicates an association between SBE and determination of starch granule size. There is a linear relationship between degree of branching and granule size [18], so that lower degree of branching resulting from mutations in the *SBE* genes may lead to synthesis of potato starch with lower average mean granule size. However, the mechanism behind inhibition of SBE and size variation of starch granules is not well understood [19]. All the high-amylopectin lines with *GBSS* mutation in *SBE*-mutated background analysed in the present study had starch granules of comparable size to those found in the native variety.

#### 3.1.2 Amylose content

Desiree had an amylose content of 27%, which is in agreement with our previous findings [13]. The highest amylose content was observed for line 104018, followed by 104006, which could be attributable to the mutations in the *SBE* genes, as previously reported [18, 19, 22–25]. This can be explained by the observation that suppressing SBE activity tended to decrease amylopectin level, consequently elevating the proportion of amylose. Inhibiting SBE activity has also been shown to reduce the branching frequency in amylopectin, hindering the incorporation of α-1,6-linkages into starch and favoring the development of elongated amylose-like chains within amylopectin [26]. Lines 104034 and 104016, despite having mutations in both *SBE*s, had similar amylose content to Desiree. This similarity could be attributable to the presence of at least one wild-type allele and one or two in-frame mutated alleles in the *SBEII* genes, whereas in lines 104018 and 104006 an in-frame mutated allele is not present. Induction of mutations in *GBSS* in the *SBE* background resulted in low-amylose lines, such as 150172 and 150183, indicating the impact of alterations in the amylose-producing enzyme, GBSS. However, line 150068 proved to be biologically interesting, as it achieved an amylose content closer to that in Desiree, despite having out-of-frame mutations in *GBSS* within an *SBE*-mutated background, as documented in our previous study [13]. Discrepancies in the amylose content of certain potato lines compared with previously reported values (12,13) may be due to variations in the production year, as observed by Jansky & Fajardo (2014) [27]. A negative correlation between amylose content and starch granule size was revealed by Pearson correlation analysis (p<0.05).

#### 3.1.3 Total phosphorus content and amounts of different forms of phosphorus

High phosphorus content in starch is a beneficial property in industrial applications. Phosphorus primarily exists as phosphate monoesters, phospholipids, and inorganic phosphate, with potato starch containing phosphorus primarily as phosphate monoesters at the C3 and C6 positions of amylopectin glucose units [28, 29]. Mutations induced in *SBE*, with or without *GBSS*, effectively increased total phosphorus content in the potato lines compared with the native variety. Specifically, mutations in *SBE* led to an average two-fold increase, with even greater increases (up to four-fold) observed in lines with mutations in both *SBE* and *GBSS* (Table 2). An exception was starch from line 150068, which exhibited only around a 0.8-fold increase compared with Desiree. High phosphorus content in starch from *SBE*-mutated lines agrees with previous findings [18, 30]. The exact mechanism behind this is not well understood, but it could be linked to molecular structure and composition alterations, which could influence the binding and incorporation of phosphorus-containing compounds within starch glucan chains. Mutations in *SBE* may indirectly affect expression or activity of other enzymes involved in starch phosphorylation, which could lead to greater accumulation of phosphorus in starch granules. Monophosphate esters associate with branched amylopectin fraction [30], so the high amylopectin content of *GBSS*-mutated 150172 and 150183 lines might be the reason for the even higher increase in total phosphorous content, in agreement with findings by Kozlov, Blennow, Krivandin, & Yuryev (2007) [31].

**Fig 1:**
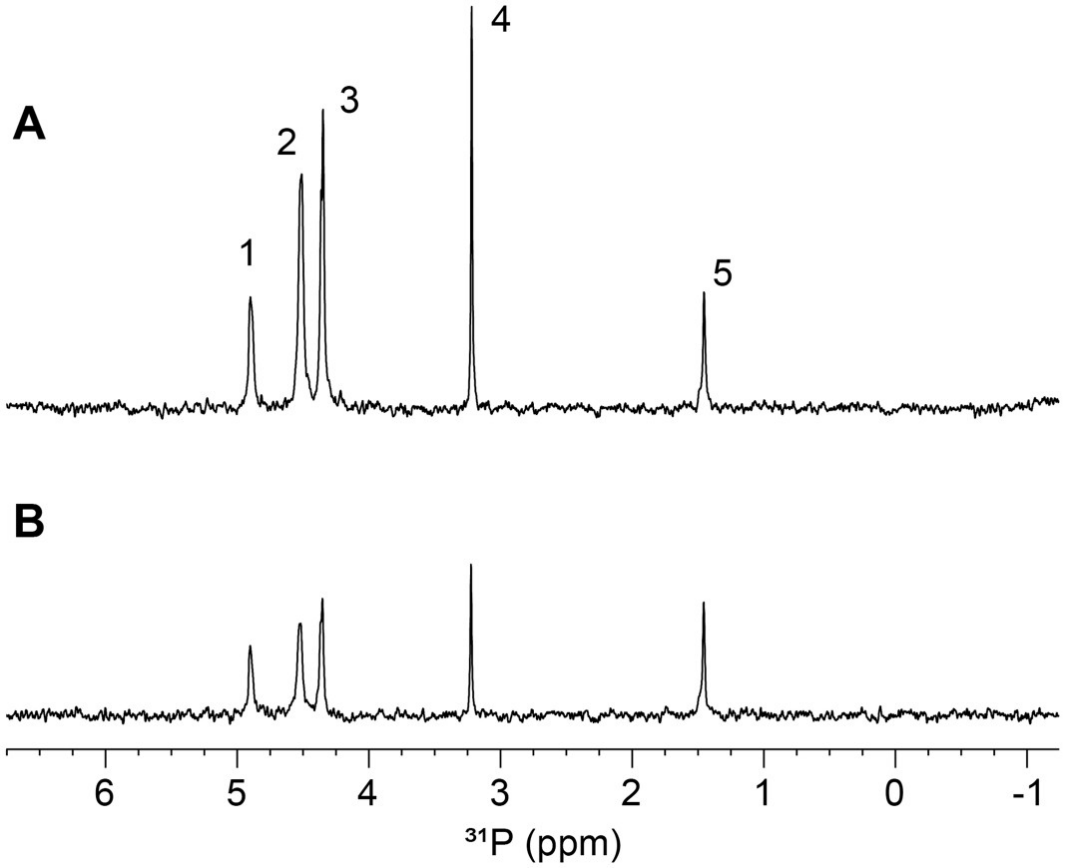
Representative ^31^P NMR spectra of α-amylase-treated starch from (A) potato line 150068 and B) native variety Desiree. Signals were assigned as (1) C3-bound phosphate, (2) C6′-bound phosphate, (3) C6″-bound phosphate, (4) free phosphate, and (5) phospholipid.

The amounts of different phosphorus forms in potato starch, as determined by NMR spectroscopy, are shown in Table 2. Fig 1 depicts the ^31^P NMR spectra of α-amylase-treated starch. In all samples, the absolute amount (g/kg) of C6-bound phosphate was consistently higher than that of C3-bound phosphate. The C3/C6 ratio was around 0.2 in starch from mutated lines except that from 150183, where it was similar to that in Desiree (0.3). Other studies have also found that C6-bound phosphate monoesters dominate in potato starch [32–34].

Analysis of the proportion of starch-attached phosphates to total phosphorus revealed that mutated lines generally had a higher proportion of their phosphorus attached to starch (around 80%) than Desiree (69%). Exceptions were seen in lines 150068 and 104018, with proportions of 75% and 73%, respectively. We observed two signals for C6-substituted phosphate, which we denoted as C6′ and C6″. A 31 P-DOSY experiment conducted to estimate the diffusion coefficient revealed that the more downfield C6′ signal originated from oligosaccharides of similar length to the C3-bound phosphate species, whereas the C6″ signal was from a smaller oligosaccharide, possibly a trisaccharide (S1 Fig). The mutated starch samples showed increased levels of inorganic phosphates, 1.2- to 3.9-fold higher than in Desiree. Pearson correlation analysis revealed a significant correlation (r = 0.94, p<0.05) between absolute amount of inorganic (free) phosphate and total starch-bound phosphate, indicating direct involvement of inorganic phosphate in starch metabolism. The phospholipid levels across all samples were rather consistent (0.09–0.14 g/kg).

#### 3.1.4. Molecular structure of granular starch

The molecular structure of the various starch types was investigated at two levels: chain length distribution of de-branched starch and internal chain length distribution of de-branched β-LDs.

**Fig 2:**
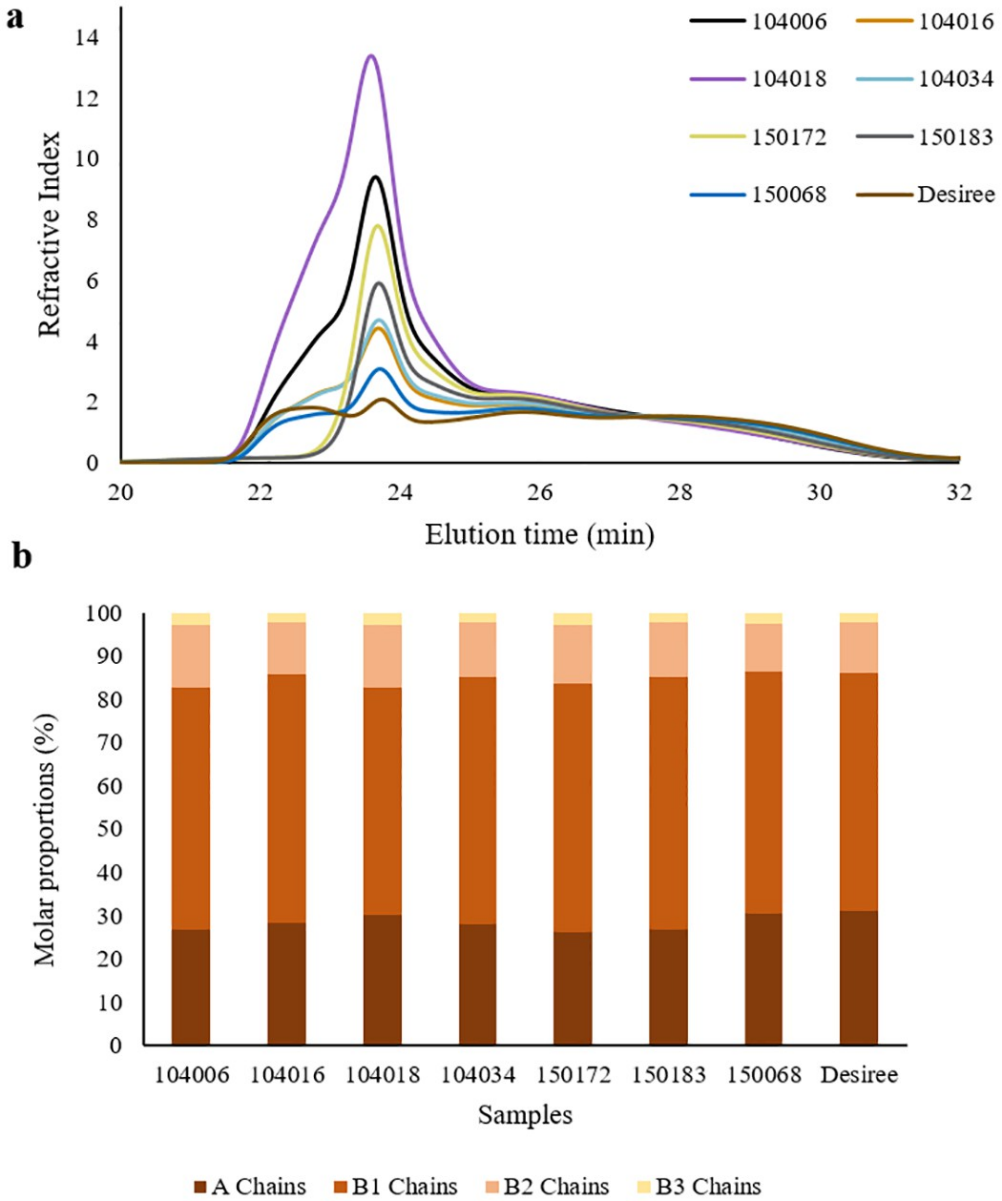
Chain length distribution of starch analysed (a) by HPSEC on a relative weight basis after normalising for amylopectin peak area 25–32 min and (b) by HPAEC on a relative molar basis with degree of polymerisation (DP) 6–50. In HPAEC analysis, amylopectin chains of de-branched starch were categorised according to Hanashiro, Abe, & Hizukuri (1996) [35] as A chains (DP 6–12), B1 chains (DP 13–24), B2 chains (DP 25–36), and B3 chains (DP ≥37).

*3*.*1*.*4*.*1 De-branched chain length distribution*. The chain length distribution of de-branched starch was examined using HPSEC and the chromatogram obtained is shown in Fig 2a. The chains eluted after 25 minutes represent the amylopectin fraction, while those eluted before 25 minutes represent the predominant amylose fraction. Significant modifications to the chain length distribution of amylose chains occurred, depending on the type of mutation, as explained in previous studies [12, 13]. Starch from lines with mutations in *SBE*s showed a prominent peak in short-chain amylose fraction eluting at around 24 min. Mutations in *GBSS* in an *SBE*s mutated background resulted in loss of the amylose long chain category, but the affected lines still had comparatively high levels of amylose short chains, which eluted just before 24 min. An exception was line 150068, which had chain length distribution close to the native variety but with elevated levels of the short amylose chain category.

A notable change in chain length distribution of amylopectin compared with the native variety was observed in starch from all mutated lines except 150068. Interestingly, the amylopectin chain length distribution exhibited a systematic pattern, correlating with the type of mutations present, as explained below. The *SBE*-mutated lines 104018 and 104006, possessing similar types of allelic mutations, showed almost identical amylopectin chain length distribution. The lines 104034 and 104016, with similar allelic mutations, also exhibited a similar amylopectin chain length distribution pattern. Additionally, starch from the *GBSS*-mutated lines 150172 and 150183 showed similar amylopectin chain length distribution. Segmenting the chromatogram into different fractions, i.e., 25–26, 26–27.4, 27.4–30 30–32 min, revealed variations in the amylopectin chain categories compared with Desiree (Table 3). This analysis was supplemented by HPAEC analysis, which enabled high-resolution examination of chain length distribution (Fig 2b, S2 Fig). Abundance of various amylopectin chain fractions, as determined by HPSEC and HPAEC, is presented in Table 3.

**Table 3:** Abundance of different categories of amylopectin chains in potato lines as analysed by HPSEC and HPAEC.

Potato line	De-branched starch								De-branched amylopectin β-limits		
	HPSEC analysis				HPAEC analysis				HPSEC analysis		
	25-26min	26–27.4 min	27.4–30 min	30–32 min	A chains	B1 chains	B2 chains	B3 chains	Long chains (25–26.2 min)	Intermediate chains 26.2–28.2 min	Short chains 28.2–32 min
104006	280±1^b^	317±1 ^a^	345±1 ^g^	57±2^c^	26.7±0.5^cd^	56.0±0.3^b^	14.5 ±0.3^ab^	2.8±0.4^a^	468±1^a^	383±2^cd^	150±3^f^
104016	234±3^f^	285±2^f^	400±0.1^c^	81±4^e^	28.2±0.4^b^	57.6±0.2^a^	12.1±0.5^cd^	2.2±0.1^a^	415±0.2^d^	390±2^ab^	195±1^c^
104018	289±2^a^	312±1 ^b^	334±1^h^	64±2^de^	30.1±0.7^a^	52.6±0.7^c^	14.6±1^a^	2.7±0.2^a^	468±5^a^	378±0.1^d^	154±5^f^
104034	241±2^e^	290±1^e^	391±1^d^	78±2^c^	28.0±0.01^bc^	57.3±0.05^a^	12.6±0.2^bcd^	2.1±0.1^a^	427±0.1^c^	390±3^ab^	183±3^d^
150172	273±1^c^	305±1 ^c^	357±1^f^	65±1^de^	26.2±0.2^d^	57.5±O.2^a^	13.5±0.4^abc^	2.7±0.4^a^	459±1^a^	380±1^d^	160±1^ef^
150183	258±0.1^d^	297±0.2^d^	376±0.1^e^	69±0.04^d^	26.90.1^bcd^	58.2±0.2^a^	12.5^c^±0.1^d^	2.4±0.1^a^	444±3^b^	387±0.03^bc^	168±3^e^
150068	215±1^g^	272±0.3 ^g^	416±0.1^b^	98±1^b^	30.6±0.2^a^	55.7±0.05^b^	11.2±0.3^d^	2.5±0.04^a^	391±3^e^	392±1^ab^	216±4^b^
Desiree	198±1^h^	262±1 ^h^	434±0.1^a^	106±2^a^	30.9±0.4^a^	55.3±0.3^b^	11.4±0.06^d^	2.4±0.07^a^	369±1^f^	394±0.1^a^	238±1^a^

In HPSEC analysis, different categories of amylopectin chain fractions from de-branched whole starch and β-limits were categorized based on different elution times. In HPAEC analysis, amylopectin chains of de-branched starch were categorized according to Hanashiro, Abe, & Hizukuri, (1996) [35] as A chains (DP 6–12), B1 chains (DP 13–24), B2 chains (DP 25–36), and B3 chains (DP ≥37). Values within cells with different superscript letters differ significantly (ANOVA, α = 0.05). Values following the mean represent the standard deviation (SD).

**Fig 3:**
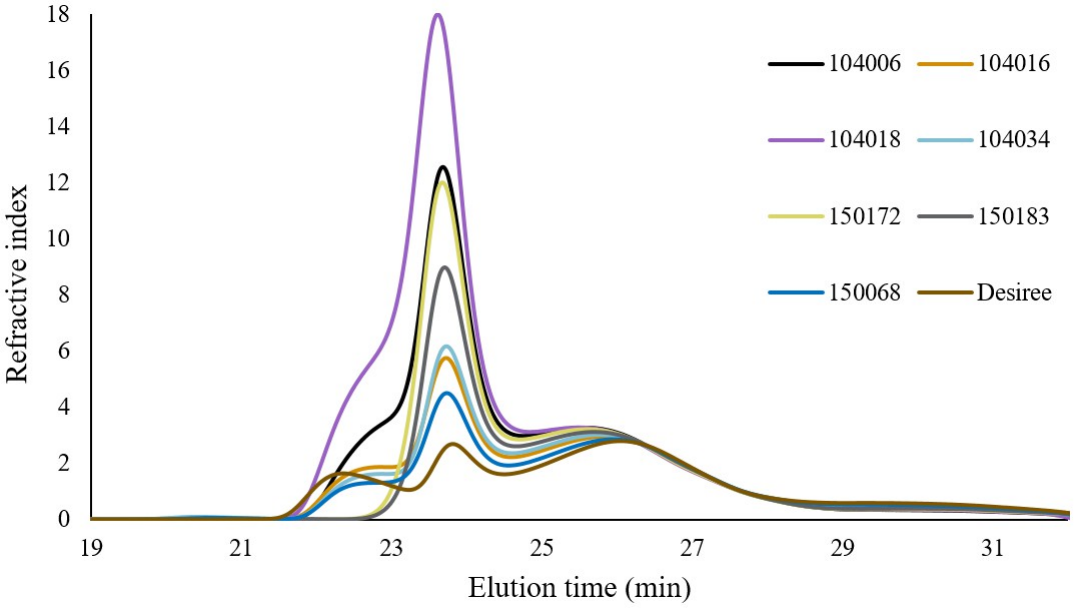
Chain length distribution of β-limit dextrins analysed by HPSEC on a relative weight basis after normalizing for amylopectin peak area 25–32 min.

*3*.*1*.*4*.*2*. *Internal chain length distribution*. Internal chain length was determined by examining the chain length distribution of de-branched β-LDs using HPSEC, as illustrated in Fig 3. The HPSEC chromatogram for de-branched β-LDs closely resembled that of de-branched whole starch (Fig 2a), encompassing chains originating from both the amylose (eluted before 25 min) and amylopectin (eluted after 25 min) chain categories. Similarly to de-branched whole starch (Fig 2a), the chain length distribution of de-branched β-LDs revealed grouping of the potato lines depending on mutation type. Potato lines 104018 and 104006 had an almost identical distribution of internal chain lengths, while potato lines 104016, 104034, and 150172, 150183 displayed a somewhat similar distribution. The internal chain distribution of 150068 closely resembled that of starch from Desiree. In samples from lines with mutations in *GBSS* within an *SBE* background, very long internal chains originating from the amylose fraction (eluting between 22–23 min) were absent. The peak around 24 min, corresponding to short internal chains from amylose, dominated in all mutated samples, unlike in Desiree. For further analysis of amylopectin internal chains, the chromatogram was segmented into different fractions, i.e., 25.0–26.2, 26.2–28.2, and 28.2–32 min, which were denoted short, intermediate, and long internal amylopectin chain fractions, respectively (Table 3). All mutated lines exhibited a higher proportion of long internal amylopectin chains and a lower proportion of short amylopectin internal chains compared with Desiree. Pearson correlation analysis indicated a significant positive correlation (p<0.05) between the internal short chain category and the A chain category, and a significant negative correlation (p<0.05) with the B2 chain category of amylopectin. A significant negative correlation (p<0.05) between intermediate-size internal chains and the B3 chain category of amylopectin was also observed. Interestingly, certain correlations between different internal chain categories and starch phosphorylation were identified. Specifically, a clear positive correlation between the long internal chain category and the absolute content of starch-attached phosphate, encompassing both C3 and C6-bound phosphates was observed. Conversely, a negative correlation was evident between the short internal chain category and the content of starch-attached phosphate. Overall, proportion of the short and intermediate internal chain categories demonstrated a negative correlation with total phosphorus content, while internal long chain fraction exhibited a positive correlation.

**Fig 4:**
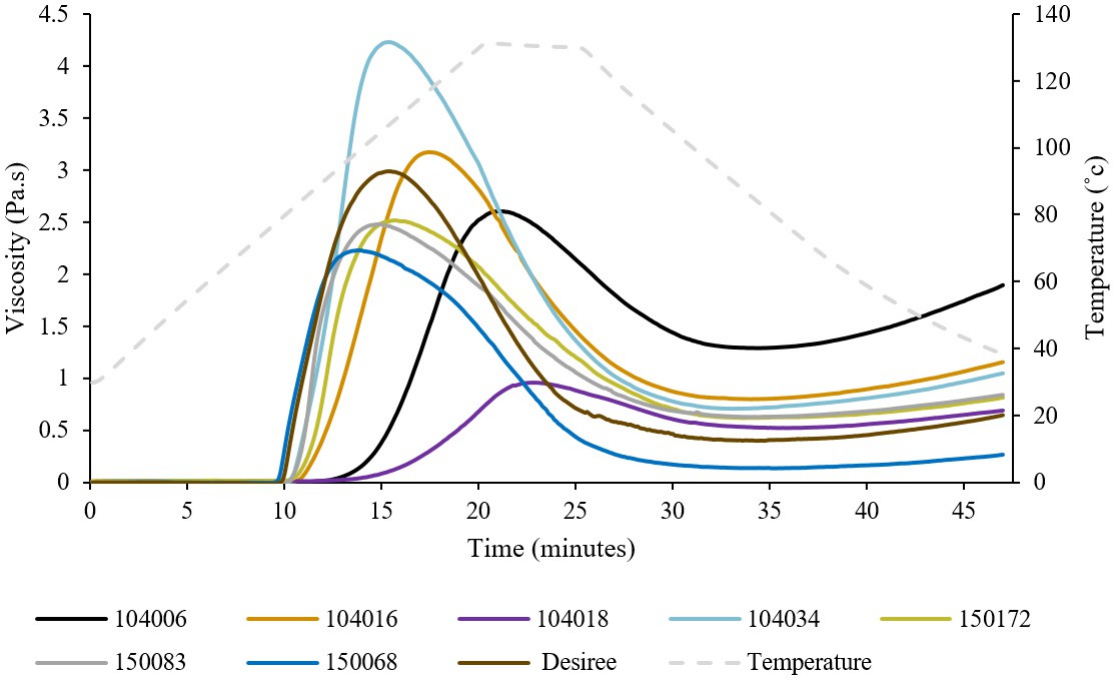
Pasting curves for starches from different potato lines obtained during a heating-cooling cycle (30-130-38 °C).

*3*.*1*.*4*.*3 Starch pasting properties*. Pasting curves obtained for different potato starch samples revealed clear differences in pasting behavior between samples with different mutations, but did not reveal distinct associations with the type of mutation (Fig 4). This lack of apparent association could be attributable to individual variations at allelic levels influencing the molecular and architectural properties of the starch, and thus the pasting behavior. Pasting parameters calculated using Trios software are shown in Table 4. Pasting temperature denotes the temperature at which the viscosity increase started due to absorption of water and loss of starch structure. Peak viscosity refers to the maximum viscosity achieved when granule swelling is at its peak. This is followed by shear-thinning, reducing viscosity to a minimum. Breakdown viscosity indicates the percentage decrease in viscosity from peak to minimum viscosity, and serves as an indicator of starch paste resistance to heat and shear forces, with lower values suggesting improved heat stability. Viscosity increases upon cooling, resulting in final viscosity, with the difference between minimum and final viscosity referred to as setback viscosity, which is attributable to amylose forming a gel network matrix [36].

**Table 4:** Pasting data for the potato starch samples.

Potato line	Pasting temperature (±0.7) (°C)	Peak viscosity (±0.06) (Pa.s)	Time at peak viscosity (±0.3) (min)	Minimum viscosity (±0.02) (Pa.s)	Breakdown viscosity (±1.4) (%)	Final viscosity (±0.02) (Pa.s)	Setback viscosity (±0.01) (Pa.s)
104006	103.7^b^±1.1	2.6^c^±0.2	20.7^b^±0.4	1.3^a^±0.01	50.6^d^±4.0	1.9^a^±0.01	0.6^a^±0
104016	88.1^c^±0.3	3.2^b^±0.02	17.1^c^±0.6	0.8^b^±0.01	74.8^c^±0.3	1.2^b^±0.01	0.4^b^±0.01
104018	112.2^a^±1.3	1.0^e^±0	22.7^a^±0.07	0.5^e^±0	45.8^d^±0	0.7^e^±0	0.2^e^±0
104034	85.2^de^±0.7	4.2^a^±0	15.4^d^±0.2	0.7^c^±0.02	83.4^b^±0.5	1.0^c^±0.04	0.3^b^±0.01
150172	86.6^cd^±0	2.5^c^±0	15.9^d^±0.07	0.6^d^±0.01	75.4^c^±0.6	0.8^d^±0.01	0.2^de^±0.01
150183	83.9^e^±0.1	2.5^c^±0.03	15.0^de^±0	0.6^d^±0.01	74.6^c^±0.3	0.8^d^±0.01	0.2^cd^±0.01
150068	78.5^f^±0.1	2.2^d^±0.01	13.9^e^±0.07	0.1^g^±0.03	94.2^a^±1.2	0.3^f^±0.03	0.1^f^±0
Desiree	79.3^f^±0.1	3.0^b^±0.01	15.4^d^±0.2	0.4^f^±0.01	86.6^b^±0.4	0.6^e^±0.01	0.3^c^±0.02

Values within cells with different superscript letters differ significantly (ANOVA, α = 0.05). Values following the mean represent the standard deviation (SD)

With the exception of 150068, starch from all mutated lines exhibited higher pasting temperatures than starch from the native variety, Desiree. Sample 104034 displayed the highest peak viscosity value, followed by 104016. Starch from the other *SBE*-mutated lines, 104006 and 104018, exhibited the lowest peak viscosities. Starch from all amylopectin lines with mutations in *GBSS* within an *SBE* background also demonstrated lower peak viscosity values compared with Desiree. However, starch from all mutated potato starch samples except 150068 displayed higher minimum viscosities than Desiree. There was no discernible pattern in breakdown viscosity attributable to mutation type. The highest final and setback viscosities compared with Desiree were exhibited by *SBE*-mutated lines, followed by *SBE+GBSS* mutated lines, with the exception of 104018 and 150068.

The pasting properties of starch are known to be influenced by its molecular structure, composition, and granular features [36–40]. In the present study, we identified several correlations (p<0.05) between the molecular and compositional properties of starch and its pasting characteristics. Pasting temperature exhibited a clear connection to molecular structure features, displaying positive correlations with the B2 chains of amylopectin and with the long unit chain categories eluted before 27.4 min in HPSEC analysis. Pasting temperature also showed positive correlations with the long internal chain category of amylopectin and negative correlations with intermediate and short internal chain category. This agrees well with existing knowledge that granule swelling is hindered by long amylopectin chains, which delay the onset of pasting in starch due to their role in maintaining granular integrity [38]. Moreover, pasting temperature exhibited a negative correlation with starch granule size and a positive correlation with phospholipid amount.

Negative correlations were observed between peak viscosity and the B3 chain category of amylopectin, in agreement with previous findings [38]. The temperature at peak viscosity displayed positive correlations with amylose content, the B2 chain category of amylopectin, and the amylopectin chain categories that eluted before 27.4 minutes in HPSEC analysis. As reported by Chen et al. [36] and Jane et al. [37], the positive correlation between temperature at peak viscosity and long amylopectin chains and amylose content is likely attributable to the fact that long amylopectin chains and amylose in starch restrict granule swelling. This in turn delays the time taken to reach peak viscosity, causing it to occur at a higher temperature. Negative correlations were observed between temperature at peak viscosity and starch granule size, the medium chain category of amylopectin internal chains, and the chain category of amylopectin that eluted after 27.4 minutes in HPSEC analysis.

Breakdown viscosity (%) exhibited positive correlations with short de-branched amylopectin chain categories that eluted after 27.4 min in HPSEC analysis and with medium and short chain categories of amylopectin internal chains. Additionally, there was a positive correlation with starch granule size, which is in agreement with findings by Singh et al. [38] and Zaidul et al. [39]. There were negative correlations between breakdown viscosity with the B2 amylopectin chain category and the long internal chain category of amylopectin. Starch with longer amylopectin chains may experience less swelling, resulting in a reduced degree of disintegration and, consequently, lower breakdown viscosity [38]. Hence, starches with high swelling capacity tended to be more susceptible to breakage, resulting in a notable decrease in viscosity after reaching peak viscosity. There was also a negative correlation between breakdown viscosity and phospholipid content.

### 3.2. Thermal properties

Starch thermal properties determined using DSC to assess gelatinization and retrogradation parameters are shown in Table 5.

**Table 5:** Gelatinization and retrogradation properties of starches from different potato lines.

Potato line	Gelatinization parameters					Retrogradation parameters	
	*T*_*o*_ (°C)	*T*_*p*_ (°C)	*T*_*e*_ (°C)	*T*_*e*_*-T*_*o*_ (°C)	ΔH (J/g)	*T*_*p*_ (°C)	ΔH (J/g)
104006	67.4^a^±0.6	76.4^a^±0.3	86.5^a^±0.4	19.1^a^±0.2	15.5^c^±0.3	73.4^ab^±1.0	7.0^e^±0.3
104016	66.2^bc^±0.01	72.9^cd^±0.1	1.6^c^±0.04	15.4^d^±0.05	19.5^b^±0.1	70.3^bc^±0.9	9.0^bcd^±0.2
104018	67.3 ^ab^±0.4	75.3^b^±0.1	85.9^a^±0.05	18.6^ab^±0.5	11.4^d^±0.4	70.3^bc^±1.8	6.7^e^±0
104034	65.0^d^±0.01	71.4^e^±0.2	1.5^c^±0.01	16.5^c^±0	20.2^b^±0.9	71.0^bc^±0.3	9.4^abc^±0
150172	65.3^cd^±0.2	73.3^c^±0.3	83.2^b^±0.3	17.9^b^±0.1	14.3^c^±0.04	75.0^a^±0.3	7.8^de^±0.4
150183	65.6^cd^±0.2	72.2^de^±0.3	81.3^c^±0.3	15.8^d^±0.04	15.7^c^±0.4	73.3^ab^±1.2	8.5^cd^±0.6
150068	63.1^e^±0.03	68.0^f^±0.04	5.6^e^±0.06	12.4^f^±0.04	19.2^b^±0.3	70.2^bc^±0.3	9.8^ab^±0.3
Desiree	63.0^e^±0.2	68.7^f^±0.2	6.7^d^±0.07	13.7^e^±0.15	22.4^a^±0.3	67.9^c^±0.2	10.4^a^±0.1

*To*—onset temperature, *Tp—*peak temperature, *Te—*end set temperature, *ΔH*–enthalpy change (calculated as J/g amylopectin). Values within columns with different superscript letters differ significantly (ANOVA, α = 0.05). Values following the mean represent the standard deviation (SD)

The gelatinization onset temperature of the starches (*T*_*o*_) ranged from 63 to 67 °C, while the peak temperature was within the range 68–76°C and the endset temperature of gelatinization (*T*_*e*_) varied from 76 to 86 °C. Compared with Desiree, *T*_*o*,_ *T*_*p*_ and *T*_*e*_ were significantly higher (p<0.05) in starch from all mutated lines except line 150068. The highest gelatinization temperatures and temperature range (*T*_*e*_*-T*_*o*_) were observed for high-amylose lines 104006 and 104016. Elevated gelatinization temperature in *SBE*-mutated lines aligns with findings in previous studies [13, 17, 41].

The broader range of gelatinization temperatures (*T*_*e*_*-T*_*o*_) of starch from all mutated lines (except 150068) suggests the presence of more heterogeneous crystals, as discussed by [42]. An interesting observation was that despite mutations in both *GBSS* and *SBE*s, line 150068 produced less heterogeneous crystals than even the native variety, Desiree.

The gelatinization temperatures aligned well with the pasting parameters, with positive correlations (p<0.05) identified between gelatinization temperature and pasting temperature, and between gelatinization temperatures and time taken to reach peak viscosity. Furthermore, gelatinization temperatures demonstrated positive correlations with minimum viscosity values and final viscosity values, and negative correlations with percentage breakdown viscosities.

These correlations between gelatinization and pasting parameters in starch are likely governed by molecular structure, compositional, and granular-level differences between the potato starch samples. Several correlations (p<0.05) were observed between the thermal properties of starch, molecular structure, composition, and granule size, as explained below. Gelatinization temperatures exhibited positive correlations with the amylopectin chain categories of B2 (from HPAEC analysis) and with the long chain categories eluted before 27.4 min in HPSEC analysis. Moreover, gelatinization temperatures showed positive correlations with long internal chains of amylopectin and negative correlations with intermediate and short internal chains. According to Zhu (2018) [43], amylopectin characterized by longer internal chains tends to result in a more organized arrangement of double helices within the granules, contributing to enhanced thermal stability. This could explain the elevated gelatinization temperatures observed for samples with a comparatively higher proportion of long internal amylopectin chains. However, we observed a negative correlation between *ΔH* and the B2 and B3 chains of amylopectin, as well as the long internal chains of amylopectin, suggesting a lower double helix content in starch as amylopectin chain length increases. Additionally, robust negative correlations were found between *ΔH* and both total phosphorus content and C6-bound phosphate content, indicating a negative effect of phosphorus on degree of crystallinity. This contradict findings by Lu, Donner, Yada, & Liu (2012) [44] of an increase *ΔH* with an increase in phosphorus content in potato starches. In agreement to findings of several other studies [45–47], negative correlations between gelatinization temperatures and starch granule size were observed. A negative correlation between granule size and *Te-To* was observed, indicating the presence of more crystalline defects in large granules, where the disintegration of the granular structure (gelatinization) initiates at lower temperatures [47].

Retrogradation refers to the process whereby glucan chains within gelatinized starch undergo re-association and recrystallization [48]. The crystal melting of retrograded amylose occurs at a higher temperature (130–160°C) than that of amylopectin [49]. Therefore, when examining starch retrogradation parameters, the primary focus in the present study was on retrograded amylopectin. The assessment of starch retrogradation was based on the peak temperature of crystal melting (*T*_*p*_), coupled with the enthalpy change in retrograded starch (*ΔH*) following storage at 4°C. The *T*_*p*_ value of the retrograded starch was within the range 70–75°C, while the *ΔH* value ranged from 7 to 10 J/g. All mutated starches exhibited higher *T*_*p*_ values and lower *ΔH* values compared with Desiree. Intriguing correlations between retrogradation parameters and the molecular structural and compositional properties of starch were observed. The *T*_*p*_ of crystal melting, which is an indication of crystal perfection of retrograded starch, displayed a positive correlation with total phosphorus content and absolute amount of total starch-bound phosphate groups. It exhibited negative correlations with the A chain category of amylopectin, the short internal chain category of amylopectin, and the short amylopectin unit chain category eluted after 27.4 min in the HPSEC chromatogram.

The enthalpy change (*ΔH*) of retrogradation also showed a negative correlation with the proportions of long amylopectin unit chain categories of B2 and B3, with those eluted before 27.4 min in the HPSEC chromatogram, and with the proportion of the long internal amylopectin chain category. As enthalpy change corresponds to the melting of double helices, the *ΔH* value of retrograded starches serves as an indicator of the extent of retrogradation [48]. The negative relationship observed between long internal chains and retrogradation *ΔH* is in agreement with findings by [17]. The longer the internal amylopectin chain lengths, the lower the density of amylopectin, resulting in lower probability of molecular rearrangement to form retrograded crystals.

### 3.3. Properties of starch films

#### 3.3.1. Optical properties of starch films

**Fig 5:**
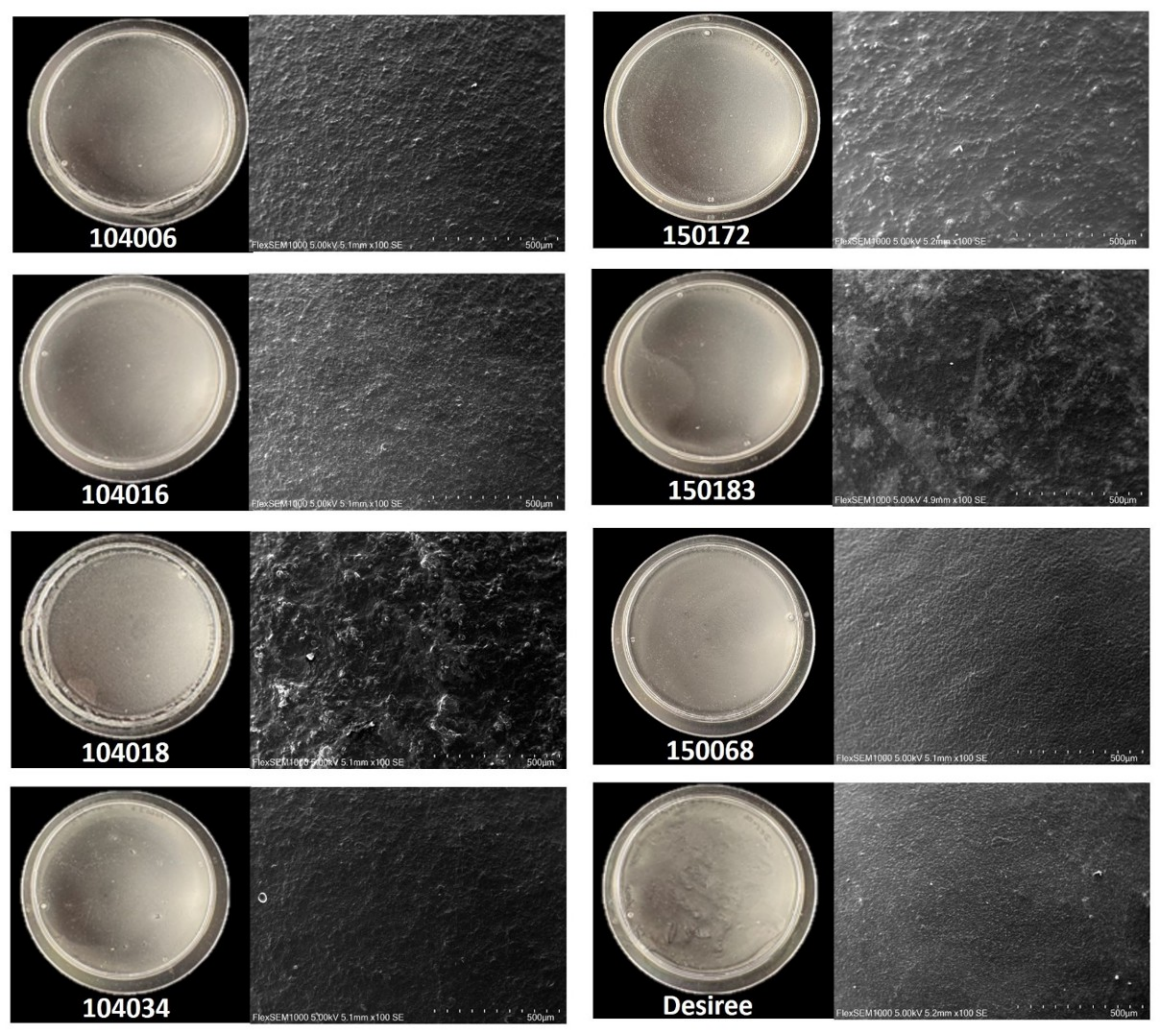
Digital images (left) and corresponding SEM images (right) depicting films from various potato lines.

All potato starch samples were successfully cast into thin films. These revealed intriguing variations in their morphological characteristics, as seen in digital images and SEM micrographs (Fig 5). Films made from starch of line 104018 displayed the roughest film surface compared with Desiree, as observed in the SEM micrograph. Notably, this specific potato starch also generated a film surface with the highest number of weak points, occurring during the film drying stage. This could be due to the high amylose content of the starch from line 104018, which could form aggregates upon casting of the film [4]. Starch from line 150068 produced the most morphologically similar film to Desiree, exhibiting smooth film surfaces. This starch type also had the most similar molecular structural, compositional, thermal and pasting properties to Desiree of all the studied lines.

#### 3.3.2 Tensile properties

Young’s modulus and elongation at break of different film samples are summarized in Table 6. No significant differences associated with the type of mutation were found, possibly due to the high standard deviations in tensile properties between film replicates. This variability can be attributed to the films’ high sensitivity to environmental variations during production across different replicates. The film samples showed differing elastic and viscoelastic tensile behaviors. The greatest deformation was seen for film from line 150172 starch, with the highest strain of 21% followed by 104034. Samples with lower Young´s modulus (<1000 MPa) (104006, 104018, 150068, 150172) could achieve >10% elongation before failing. Films from Desiree, 104016 and 150183 starches failed close to 5% strain. A negative correlation was found between Young’s modulus and the long chain fraction of amylopectin (DP≥37), contradicting previous findings [4]. Furthermore, unlike in previous studies [4, 50], there was no correlation between high amylose lines and improved mechanical properties of the films. This could be due to the high standard deviation values obtained for the samples, caused by low reproducibility of film preparation.

**Table 6:** Summary of the mechanical and thermal properties.

Potato line	Mechanical properties		Thermal properties	
	E (MPa)	ε_break_ (%)	*T*_*o*_ (°C)	*T*_*p*_ (°C)
104006	724.6±103.5	15.3±3.5	280.8^b^±1.9	296.7^c^±0.2
104016	1280.2±190	4.9±2.5	283.7^b^±1.7	300.0^b^±0.3
104018	419.3±274	10.6±4.9	274.4^de^±1.3	289.3^e^±0.4
104034	1043.1±754	18.1±16.1	280.4^bc^±2.0	296.6^c^±0.5
150172	560.2±197	21.3±1.5	273.6^e^±3.0	290.4^e^±0.5
150183	1060.3±311	4.5±4.0	277.0^cd^±1.1	293.1^d^±0.8
150068	598.0±7.52	12.0±2.4	274.8^de^±1.5	294.0^d^±1.4
Desiree	1213.9±135.7	4.6±1.6	288.7^a^±0.9	306.5^a^±0.5

E—Young´s modulus, ε_break_- strain at break, T_*o*_—onset temperature, T_*p*_—peak temperature. Values within columns with different superscript letters differ significantly (ANOVA, α = 0.05). Values following the mean represent the standard deviation (SD).

#### 3.3.3. Thermal properties

**Fig 6:**
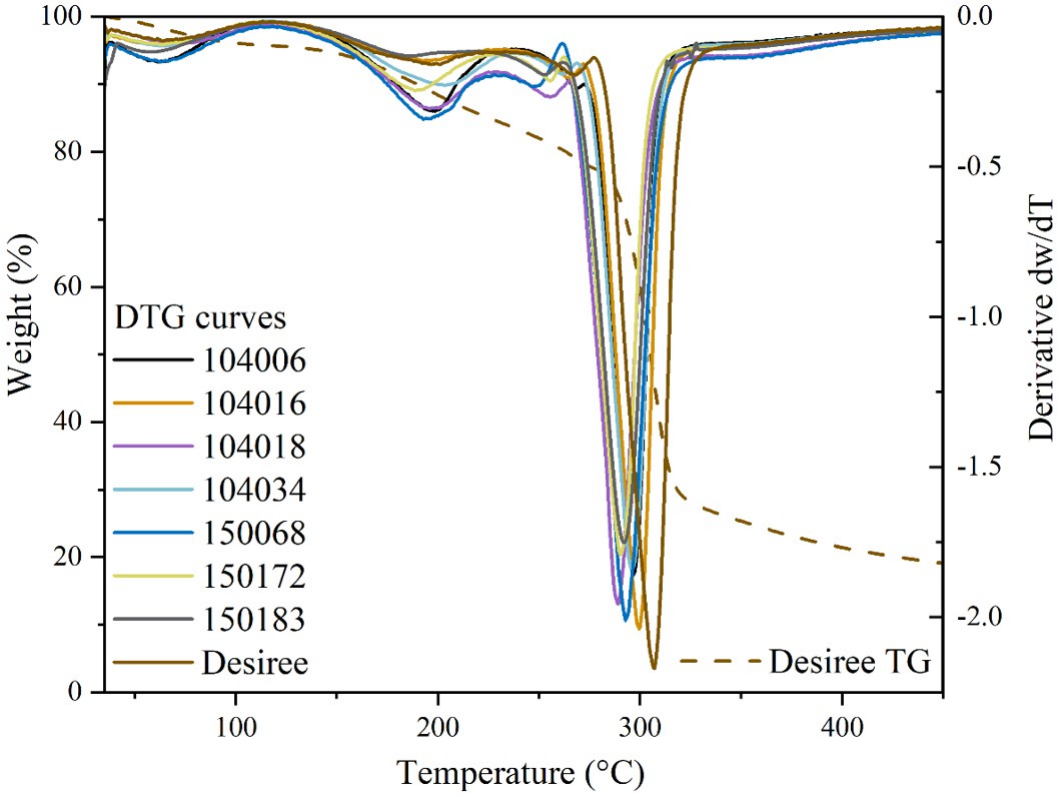
First derivative curves (dw/dT) of different potato film samples and example of a thermal gravimetry (TG) curve of film prepared from desiree.

Thermal properties of the starch films and their thermal degradation were analysed by TGA. Table 6 summarizes the onset and peak temperature values of the films, which differed significantly. Thermographs and their first derivatives (dw/dT) (Fig 6) fell into three significant areas where different components present in the samples evaporated and degraded. Up to 100°C, samples lost 3.0–4.08% of their total weight due to evaporation of free water from the films. The next degradation step, between 100 and 250 °C, reflected decomposition of glycerol and the weight loss ranged from 7.1 to 12.4%, which corresponded to the amount of glycerol added during film preparation. The DTG curves displayed a small degradation peak between 250–270°C, indicating the presence of impurities of the starch samples and causing 2.0–3.8% weight loss. Starch degradation occurred between 270 and 350 °C, where the samples lost 37.8–46.7% of their weight.

Onset temperature (*T*_*o*_) and peak temperature (*T*_*p*_) of starch degradation were significantly higher (p<0.05) for Desiree than for all mutated lines. Starches from *SBE*-mutated high amylose lines (series 104-) showed the second highest *T*_*o*_ and *T*_*p*_, except for 104018. Starch from *GBSS-*mutated high-amylopectin lines (series 150-) exhibited the lowest *T*_*o*_ and *T*_*p*_ temperatures (Table 6).

Negative correlations (p<0.05) were observed between *T*_*p*_ and total phosphorus, free inorganic phosphate, and C6’ phosphate contents. Additionally, *T*_*p*_ was positively correlated (p<0.05) with the medium and short chain fractions of internal chains, and negatively correlated with the long internal chain fraction and long amylopectin unit chains (eluted between 23–25 min in the HPSEC chromatogram).

#### 3.3.4. Oxygen barrier properties

Oxygen transmission rate (OTR) and oxygen permeability in comparison with Desiree were studied using film from 150068 starch. This line was selected for comparison owing to its interesting molecular structure similarities to Desiree, despite significant mutations. Starch from line 150068 also showed promising pasting properties, including lowest pasting temperature, shortest time to peak viscosity, and low viscosity at moderate temperatures. In fact, the low viscosity of line 150068 starch at the film casting temperature may have enhanced film formability and reproducibility, resulting in smooth surfaces.

The data obtained on oxygen barrier properties indicated that OTR (8.5 ± 1 cc/m^2^/24h) and oxygen permeability (0.3 ± 0.05 cc·mm/m^2^·24h·atm) of 150068 did not differ significantly (p>0.05) from those of Desiree (7.4 ± 0.8 cc/m^2^/24h and 0.3 ± 0.02 cc·mm/m^2^·24h·atm, respectively). Therefore, the oxygen barrier properties of starch from the mutated 150068 line were not compromised compared with those of starch from the native variety.

## 4. Conclusions

This study revealed significant impacts of induced targeted mutations in starch biosynthetic genes on the chemical and functional properties of potato starch. Mutations in *SBE*s led to smaller starch granules and higher amylose content. Total phosphorus content was elevated in starch from all mutated lines, especially those with mutations in *GBSS* in the *SBE*s mutated background. NMR spectroscopy revealed alterations in starch phosphorylation patterns in mutated lines compared with the native variety (Desiree), with mutations resulting in increased proportions of starch-bound phosphates. *SBE*-mutated lines produced starches with high proportions of long amylopectin chains, influencing the functional properties of the starch. Long amylopectin chains and elevated amylose content were associated with higher gelatinization and pasting temperatures of starch. The enthalpy change related to starch gelatinization was negatively influenced by total phosphorus content, indicating negative effects of phosphorus on starch crystallinity. However, the peak temperature of crystal melting of retrograded starch exhibited positive correlations with total phosphorus content and negative correlations with short amylopectin chains. The morphological, mechanical, thermal, and barrier properties of potato starch films derived from mutated lines exhibited interesting variations. However, no direct associations were found between starch molecular features and starch film properties, highlighting the high heterogeneity of the films and the critical role of film preparation in determining film properties. Mutated starch types appeared highly sensitive to environmental factors during film formulation. Starch from a particular potato line with a complete knockout of *GBSS* in the *SBE*s-mutated background (line 150068) had molecular and functional properties closely resembling those of Desiree and produced films with similar morphological properties to those made from Desiree starch. The similarity of molecular features between Desiree and 150068, extending to functional and film properties, represents a promising avenue for utilization of such starch types. This study provided valuable insights into the links between physicochemical and functional properties of starch, suggesting huge potential in application of mutated starch types in various industries, including packaging. Further research is needed to fully optimize industrial utilization of starches resulting from targeted mutations in potato lines.

## Supporting information

S1 Fig 31P-DOSY spectrum of α-amylase-treated starch from line 150172 (assignments as in Fig 1). The phospholipid signal did not show up in the DOSY spectrum, due to much lower diffusion coefficient (D). The DOSY experiment involved a bipolar-pair longitudinal-eddy-current delay (BPP-LED) (Bruker pulse sequence ledbpgp2s) with diffusion time (Δ) of 100 ms and effective gradient pulse duration (δ) of 4.4 ms.
(TIF)

S2 Fig Chain-length distribution of debranched starches on a relative molar basis (M%) with degree of polymerization (DP) 6–50, based on HPAEC analysis. (TIF)
