# Supplementary figures and images for: Impact of mutations in starch synthesis genes on morphological, compositional, molecular structure, and functional properties of potato starch
Source: PLoS One. 2024 Sep 26;19(9):e0310990. doi: 10.1371/journal.pone.0310990 (PMC11426511; doi:10.1371/journal.pone.0310990)

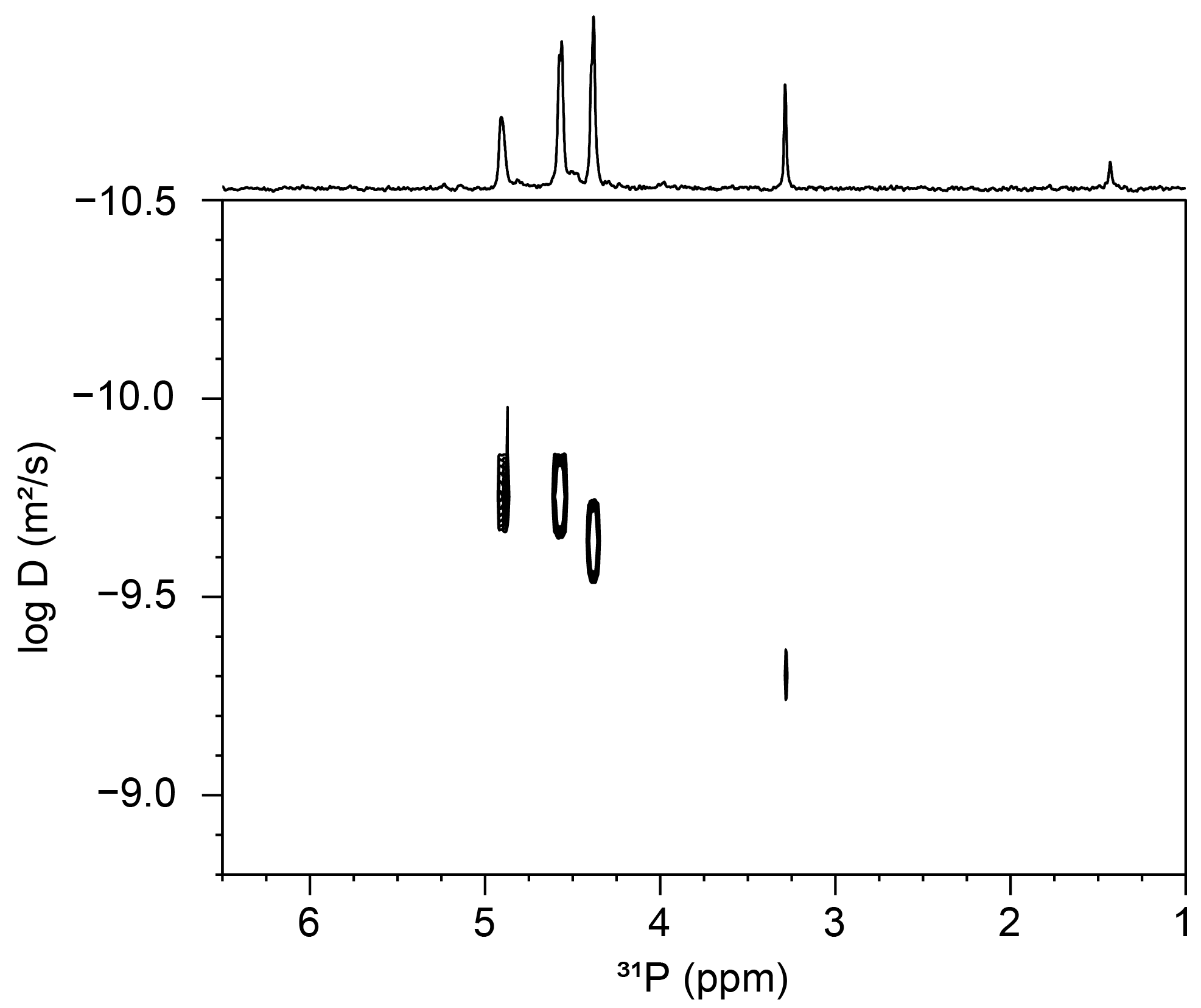

Supplement: S1 Fig — The phospholipid signal did not show up in the DOSY spectrum, due to much lower diffusion coefficient (D). The DOSY experiment involved a bipolar-pair longitudinal-eddy-current delay (BPP-LED) (Bruker pulse sequence ledbpgp2s) with diffusion time (Δ) of 100 ms and effective gradient pulse duration (δ) of 4.4 ms. (TIF) [file pone.0310990.s001.tif]

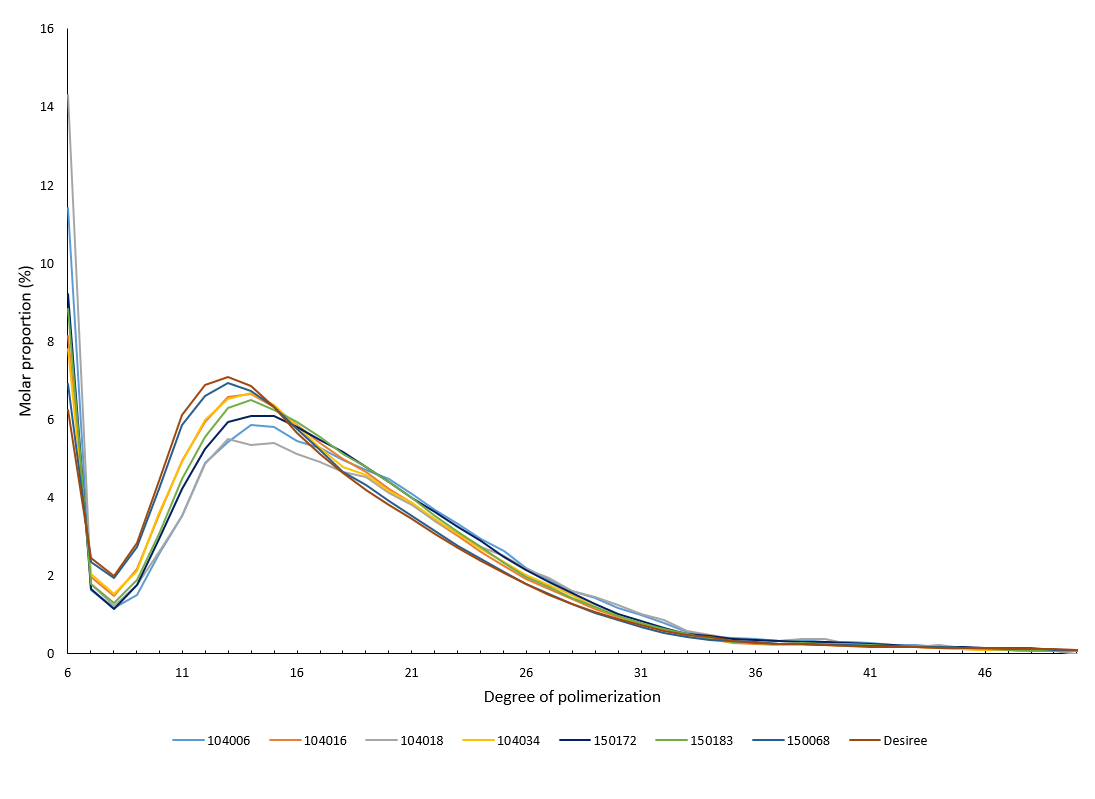

Supplement: S2 Fig — (TIF) [file pone.0310990.s002.tif]
